# Immunomodulatory and Antiprotozoal Potential of Fabricated *Sesamum radiatum* Oil/Polyvinylpyrrolidone/Au Polymeric Bionanocomposite Film

**DOI:** 10.3390/polym13244321

**Published:** 2021-12-10

**Authors:** Nawal A. Alarfaj, Musarat Amina, Nawal M. Al Musayeib, Maha F. El-Tohamy, Gadah A. Al-Hamoud

**Affiliations:** 1 Department of Chemistry, College of Science, King Saud University, Riyadh 11451, Saudi Arabia; nalarfaj@ksu.edu.sa (N.A.A.); moraby@ksu.edu.sa (M.F.E.-T.); 2 Department of Pharmacognosy, Pharmacy College, King Saud University, Riyadh 11451, Saudi Arabia; nalmusayeib@ksu.edu.sa (N.M.A.M.); galhamoud@ksu.edu.sa (G.A.A.-H.)

**Keywords:** *Sesamum radiatum* oil, bionanocomposite, polymeric film, polyvinylpyrrolidone, immunomodulatory, antiprotozoal

## Abstract

A unique morphological *Sesamum radiatum* oil/polyvinylpyrrolidone/gold polymeric bionanocomposite film was synthesized using the *S. radiatum* oil dispersed in a polymeric polyvinylpyrrolidone (PVP) matrix and decorated with gold nanoparticles (AuNPs). The chemical and physical characteristics as well as the thermal stability of the synthesized bionanocomposite film were investigated using various spectroscopic and microscopic techniques. The microscopic analysis confirmed well dispersed AuNPs in the PVP- *S. radiatum* oil matrix with particle size of 100 nm. Immunomodulatory and antiprotozoal potentials of the suggested bionanocomposite film were evaluated for lipopolysaccharide-induced BV-2 microglia and against *L. amazonensis*, *L. mexicana* promastigotes and *T. cruzi* epimastigotes, respectively. The results exerted outstanding reduction of inflammatory cytokines’ (IL-6 and TNFα) secretions after pretreatment of bionanocomposite. The bionanocomposite exhibited large inhibitory effects on certain cell signaling components that are related to the activation of expression of proinflammatory cytokines. Additionally, AuNPs and bionanocomposite exhibited excellent growth inhibition of *L. mexicana* and *L. amazonensis* promastigotes with IC_50_ (1.71 ± 1.49, 1.68 ± 0.75) and (1.12 ± 1.10, 1.42 ± 0.69), respectively. However, the nanomaterials showed moderate activity towards *T. cruzi*. All outcomes indicated promising immunomodulatory, antiprotozoal, and photocatalytic potentials for the synthesized *S. radiatum* oil/PVP/Au polymeric bionanocomposite.

## 1. Introduction

Recent advances in nanomaterials are due to the development of various engineered nanoparticles, particularly noble metal nanoparticles such as (Silver, gold, platinum, etc.), which are considered as a distinctive group of nanomaterials with specific physical and chemical characteristics [1]. Efficiently, noble metal nanoparticles have been widely utilized for different biomedical applications [2,3]. Nowadays, among these noble metals, gold nanoparticles (AuNPs) have gained extensive attention due to their unique small size, high reactivity with bioactive molecules, biocompatibility, stability over a wide temperature range, and their ability to decorate and exploit various biomedical uses [4]. Gold nanoparticles are applied in the diagnosis as well as in the treatment of cancer [5], DNA-AuNPs’ assemblies as sensing systems [6], protein-based recognition systems to enhance the immunoassays [7], drug delivery systems [8], antibacterial potential against various pathogens [9], antioxidants [10], and photocatalysis [11].

Conventional chemical procedures for the synthesis of AuNPs require hazardous and toxic compounds that could result in potential human and environmental threats. Therefore, there is always a great desire to encourage the development of economic, efficient, environmentally compatible approaches to elude the use of hazardous chemicals in the synthesis of nanoparticles [12]. To overcome these obstacles, natural resources (plant extracts, bacteria, and marine algae) provide excellent products suitable for the biogenic synthesis of metal nanoparticles [13,14,15]. The advantages of fabricating a sustainable nanocomposite by incorporating the metal nanoparticles with lower dimensional polymeric matrix produce highly promising applications in food industries and biomedical uses [16,17]. With modern technologies, green-synthesized, nanocomposite-based polymers and natural oils have enormous potential in minimizing the growing environmental harm [18].

Sesame oil derived from edible seeds of *Sesamum* *radiatum* L. (Family: Pedaliaceae), a major annual crop of a naturalized flowering shrub cultivated in Asia since ancient times [19]. The oil has been positioned in ninth place among the top 13 oil seed crops, which is comprised of 90% of the world population of edible oils. It is highly valuable oil and has several industrial applications such as pharmaceuticals, soaps, cosmetics, perfumery, paints, insecticides, hydrogenated oil, hair oil, and medicines [20]. The oil contains a high content of unsaturated fatty acids, free from unwanted odors, and is rancidity resistant, which makes it best suited for cooking [21]. Sesame oil possesses various biological properties including antiaging, antihypertensive, lower serum lipid antibacterial, and anticancer effects [22,23]. The main essential oil components present are sesamol, sesamolin, sesamin, contain carboxylic, and phenolic functional groups that serve as potent antioxidants [24].

Nevertheless, the synthesis of oil/metal bionanocomposite is not facile but complicated. Therefore, a polymeric matrix is required as a supporting material in the preparation of a targeted bionanocomposite. Various biodegradable polymers, including polyurethane, polyvinyl pyrrolidone, polyetherimide, polyesteramide, epoxy, alkyd, polystyrene, and polyvinyl alcohol, have been used in the preparation of oil/metal bionanocomposites [25,26]. Among these, polyvinyl pyrrolidone (PVP) is a chemically non-toxic polymer containing both hydrophilic and hydrophobic groups [27]. It stabilizes and prevents the aggregation of nanoparticles due to the repulsive forces that arise from the hydrophobic carbon chains that are interacting with each other when dispersed in the solvents [28]. Due to its excellent physical and chemical properties (optical transparency, chemically inert, high mechanical stability), it is often used as an excellent binding and protective material [29]. Moreover, PVP has been exploited for many significant applications in various research areas such as biomolecules, biosensing systems, and biomedical [30,31]. PVP has also been recommended by the Food and Drug Administration as a non-toxic polymer for biomedical measurements.

There are only a few reports that have been addressed in the literature that describe the use sesame oil in the green synthesis of silver nanoparticles and their bioactivity including antibacterial, antioxidant, and anticancer potentials [32,33]. Considering the great medicinal benefits of sesame oil, AuNPs, and PVP, the present study suggests the synthesis of a polymeric sesame oil/PVP/Au bionanocomposite film and explores its biomedical potential, including immunomodulatory, antiprotozoal, and photocatalysis.

## 2. Experimental

### 2.1. Chemicals and Reagents

The chemicals and reagents in this work were purchased from Sigma Aldrich (Hamburg, Germany). All chemicals are pure grade with high purification, including tetrachloroauric acid (HAuCl_4_, 99.0%), potassium persulfate (K_2_S_2_O_8_), Polyvinylpyrrolidone (PVP), trisodium citrate dihydrate, sodium hydroxide (97.0%), phosphate buffer saline (pH 7.4), fetal bovine serum, and Dulbecco’s Modified Eagle Medium (DMEM). Different pure solvents were used, including ethanol (99.0%), tetrahydrofuran (THF, 99.9%), N,N-dimethylformamide (DMF, 99.0%), dimethyl sulfoxide (DMSO, 99.9%), anhydrous chloroform (99.8%), Radioimmuno precipitation assay buffer (RIPA), lipopolysaccharide (LPS), cell counting kit-8 (CCK-8)Water-soluble tetrazolium salt (WST-8 reagent), 1% sodium-dodecyl sulphate, Quick RNA mini kit (Zymo Research, Irvine, CA, USA), High capacity cDNA Reverse Transcription Kit (Applied Biosystems, Thermo Fisher Scientific Inc., Waltham, MA, USA), and Liver infusion tryptose (LIT) medium.

### 2.2. Botanical Material

The *S. radiatum* seeds were acquired from local outlets of Riyadh, Saudi Arabia, and were identified taxonomically at the Pharmacognosy Department, College of Pharmacy, King Saud University. The undamaged seeds were collected and selected manually.

### 2.3. Cell Culture and Parasites

BV-2 murine microglial cells were obtained from the Research Center of King Faisal Hospital, Saudi Arabia. The cells were maintained in Dulbecco’s Modified Eagle Medium (DMEM), supplemented with 1% penicillin/streptomycin and 10% fetal bovine serum (FBS). A poly-L-ornithine coated dishes were used to culture the cells. Six-well plates were applied to seed BV-2 cells and cultured for 1 day prior to the treatments. The cells were then treated with 200-fold diluted test samples and standards to evaluate their effects on the production of cytokines. Lipopolysaccharide (LPS) treatment (1.0 μg mL^−1^, *Escherichia coli* O55:B5, Sigma-Aldrich Kft., Hamburg, Germany) was used. Liver infusion tryptose (LIT) medium was used to grow *Leishmania amazonensis* (MNYC/BZ/62/M384 strain), *Leishmania mexicana* (MNYC/BZ/62/M379 strain) promastigotes, and *Trypanosoma cruzi* epimastigotes (RA strain). Parasite cultures were maintained by weekly transit between 26 °C and 28 °C. Prior to the experiment, the parasites were transited between 24 or 48 h.

### 2.4. Extraction and Purification of Sesamum radiatum Oil

**Figure 1:**
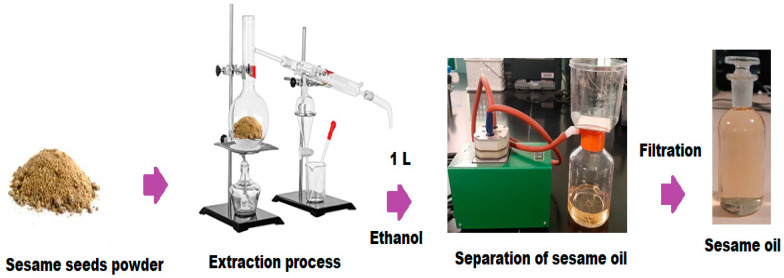
Extraction steps of sesame oil using Clavenger apparatus and 1.0 L of deionized water at room temperature.

The *S. radiatum* seeds (1000 g) were de-shelled and cleaned well with distilled water to remove the contaminants. The cleaned seeds were air dried for 7 days to free them from the remaining moisture and further oven dried at 40 °C. Approximately, 500 g of dried sesame seeds were pulverized to a fine powder using a heavy-duty grinder. The powder of *S. radiatum* seeds (250 g) was extracted in a Soxhlet apparatus using 1 L ethanol for 4 h. The extracted oil was filtered and subjected to reduced pressure in a rotary evaporator to remove the solvent. The obtained oil was dried over anhydrous sodium sulfate, transferred in capped glass vials, and stored in a refrigerator at 4 °C for further studies (Figure 1). 

### 2.5. Synthesis of Gold Nanoparticles

AuNPs were synthesized using a simple reduction method. Briefly, 20 mL of HAuCl4 (2.8 × 10^−4^ mol L^−1^) solution were heated to 100 °C then reduced by adding 3 mL of 0.5% trisodium citrate dihydrate solution. The reaction mixture was heated continuously at 60 °C for 30 min. The completion of the reaction mixture was monitored by looking for a pale purple. The change in the color from pale purple to dark purple indicates the formation of AuNPs [34].

### 2.6. Biogenic Synthesis of Sesame Oil/PVP/Au Bionanocomposite

**Scheme 1:**
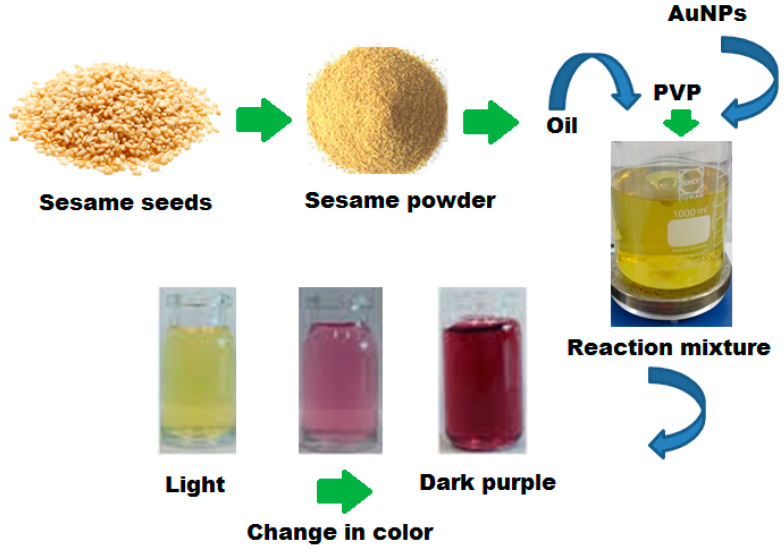
Schematic diagram of the polymeric synthesis of Sesame oil/PVP/Au bionanocomposite and gradual change in color from light yellow to dark purple.

An ultrafine polymeric sesame oil/PVP/Au bionanocomposite film was synthesized by the dropwise addition of 10 w % of sesame oil to 10% PVP solution (10 g of PVP dissolved in 100 mL of Water: THF, 98:2) under continuous overnight magnetic stirring to get a homogenous polymeric matrix. Afterwards, 5% of AuNPs was added to the polymeric solution and subjected to continuous magnetic stirring for another 6 h at room temperature [35]. The change in color from a colorless solution to a dark purple revealed the formation of bionanocomposite film. Scheme 1 represents the overview procedure for the synthesis of a polymeric sesame oil/PVP/Au bionanocomposite film.

### 2.7. GC-MS Analysis of Sesame Oil

The chemical components of sesame oil were analyzed using gas chromatography-mass spectrometry (GC-MS, Perkin Elmer Auto XL gas chromatography) joined with the ionization flame detector. The analysis was carried out using an EQUITY-5 column (60 m × 0.32 mm × 0.25 μm diameter)-fused silica capillary column. The carrier gas, H2, was adjusted at 10 psi pressure. The temperature of the column was optimized at 70 °C (2 min), followed by gradient elevation to 250 °C at 37 °C/min (10 min). The temperature of the injector and detector was optimized at 250 °C and 280 °C, respectively. The elution was directly carried out from GC to mass source and the electron ionization mode 70 eV was applied to detect the mass spectra over a scanning rate of 50–500 amu for 2 s. The active constituents of sesame oil were determined by comparing the retention time of each component with the authentic sample employing the homologous series of n-alkanes (C8–C32). Wiley, NIST, and NBS mass spectral libraries were also used to match the obtained spectral peaks [36].

### 2.8. Characterization of AuNPs and Sesame Oil/PVP/Au Bionanocomposite

Different spectroscopic methods were applied to confirm the synthesis of AuNPs and sesame oil/PVP/Au bionanocomposite. The UV-Vis spectrophotometry (Ultrospec 2100^®^ pro, Biochrom, Cambridge, UK), Fourier-transform infrared spectroscopy (FT-IR) technique within a 4000–400-cm^−1^ range, and X-ray diffraction (XRD, X-ray diffractometer, D/MAX 2500, Rigaku Corporation, Tokyo, Japan) were used to study the crystalline form of the synthesized nanoparticles. The presence of gold in the bionanocomposite was confirmed by XRD analysis at Bragg angles ranging from 10°–70°, voltage of 30 kV, and 45 mA. Dynamic Light Scattering (DLS, HORIBA, LB-550 version program) was used to study the particle size distribution. The microscopic examination was carried out by using a Transmission electron microscope (TEM, JEM-1400 Plus, JEOL, Peabody, MA, USA) and scanning electron microscope (SEM, JSM-7610F, JEOL, Peabody, MA, USA) to study the surface morphology and particle size.

### 2.9. Thermal Stability of Sesame Oil/PVP/Au Bionanocomposite

A TGA-502 thermogravimetric analyzer (Shimadzu, Tokyo, Japan) was applied to study the thermal stability of the synthesized sesame oil/PVP/Au bionanocomposite. Around 5 mg of bionanocomposite sample was heated from ambient temperature to 600 °C at a heating rate of 25 °C/min under the steady flow of nitrogen gas (50 cm^3^/min) to obtain the thermogravimetric analysis (TGA) without oxidative decomposition. The differentials of TGA values were used to obtain the TGA derivative (TGAD), which was determined by applying a forward finite difference method:

TGAD = (w_t+∆t_ − w_t_)/∆t)

where w_t+∆t_ and w_t_ represent the residual weight of test sample at t + ∆t time and t, respectively. The ∆t is the time interval for residual test sample weight reading.

### 2.10. Anti-Inflammatory Effects of AuNPs and Bionanocomposite

The Anti-inflammatory potential of AuNPs and sesame oil/PVP/Au bionanocomposite was investigated in three different experiments: LPS pretreatment for 24 h then individual sample treatment for 24 h; sample pretreatment for 24 h then LPS treatment for 24 h; and, finally, co-treated with combined LPS and test samples for 24 h. Experimental controls used were DMSO treated cells. Three concentrations of DMSO (0.05, 0.02, 0.01%) were used in the experiments with respect to the dilutions. Each experiment was performed in a humidified atmosphere at 37 °C with continuous supply of 5% CO_2_ in triplicates.

### 2.11. Cell Viability Assay

Ninety-six-well plates using 5 × 10^3^ cells/well were plated with BV-2 cells and treated with AuNPs, sesame oil/PVP/Au bionanocomposite, and standards in three dilutions (200, 500, 1000 fold) for 12 h and 24 h. The BV-2 cell viability was measured by cell viability assay using cell counting kit-8 (CCK-8) after the treatments. The cells treated with DMSO were used as controls for AuNPs and bionanocomposite-treated cells, whereas the DMSO effect on cell viability was measured against untreated cells as controls. Afterwards, 10 μL of water-soluble tetrazolium salt (WST-8) reagent were added to each well plate and incubated at 37 °C for 1 h with continuous flow of 5% of CO_2_. After 1-h incubation, 1% sodium-dodecyl sulphate (10 μL) was added to each well plate to stop the reaction. MultiSkan GO microplate spectrophotometer (Thermo Fisher Scientific Inc., Waltham, MA, USA) was used to measure the absorbance of the samples at 450-nm wavelength. The cell viability was calculated as a percentage of the total cell number with respect to the appropriate control.

### 2.12. Real-Time Polymerase Chain Reaction (PCR)

The BV-2 cells with density 3 × 10^5^ cells/well were treated in 6-well plates in a similar way as mentioned earlier. The phosphate buffer saline (PBS, pH 7.4) was used to wash the treated BV-2 cells and were collected after trypsinization. The Quick RNA mini kit was applied to extract the total RNA from each sample. The total RNA (200 ng) was used to prepare the complementary DNA using high-capacity cDNA Reverse Transcription Kit by obeying the manufacturer’s instructions. The CFX96 real-time System was applied to determine the gene expressions in 20 μL of total reaction volume, using iTaq™ Universal SYBR^®^ Green Supermix (Bio-Rad Inc., Hercules, CA, USA). After each quantitative PCR run, melting graphs was plotted to ensure that a single, specific product was amplified. The Livak (ΔΔCt) method was used to calculate the relative quantification using the Bio-Rad CFX Maestro software (Bio-Rad Inc., Hercules, CA, USA). β-actin was used as a comparative control for the expression level of the gene of interest in each sample. These relative rates of expression were then compared between untreated and treated samples. The relative rate of expression for the controls was labeled as one [37]. Controls were used to compare the mRNA expression of the treated cells. The primer sequences applied in this study are listed in Table 1.

### 2.13. ELISA Measurements

The culture media of the treated and control cells were collected after each treatment and kept in refrigerator at −80 °C until the measurements were performed. The concentration of secreted TNFα and IL-6 of the culture media was measured using TNFα and IL-6 mouse ELISA Kits (Thermo Fisher Scientific Inc., Waltham, MA, USA) following the manufacturer’s instructions [38].

### 2.14. Immunoblotting

Six-well culture plates were seeded with BV-2 cells (3 × 10^5^ cells/well) and incubated for 24 h at room temperature. After 24-h incubation, the cells were treated with the test samples. The treated BV-2 cells were collected and immediately fractionated using the Subcellular Protein Fractionation Kit (Thermo Fisher Scientific Inc., Waltham, MA, USA) following the manufacturer’s instructions. DC Protein Assay Kit (Bio-Rad Inc., Hercules, CA, USA) was applied to estimate the protein contents of each protein fraction. Fifteen μg of protein from each sample were plated onto SDS-polyacrylamide gels (10% or 12%). The protein content of the gels was moved to nitrocellulose membranes (Pall AG, Basel, Switzerland) by electro-blotting after the electrophoresis. Then, 5% non-fat dry milk in tris buffer saline (TBST, 0.1% Tween-20) was used to block the membranes at room temperature for 1 h [39]. Then, the membranes were explored with polyclonal rabbit antibodies at 4 °C overnight, according to manufacturer’s protocols: anti-NF-κB/p65 IgG (1:2000, Cell Signaling Technology Europe, Leiden, The Netherlands), anti-NF-κB/p50 IgG (1:1000, Sigma-Aldrich Kft., Budapest, Hungary), and anti-phospho-C/EBPβ IgG (1:1000, Thermo Fisher Scientific Inc., Waltham, MA, USA). β-actin (1:2000; Sigma-Aldrich Kft., Budapest, Hungary) was applied as a housekeeping control in all Western blot experiments. The secondary antibody used in the experiment was Goat anti-rabbit HRP-conjugate (1:3000; Bio-Rad Inc., Hercules, CA, USA). The detection of protein was performed using WesternBright ECL chemiluminescent substrate (Advansta Inc., San Jose, CA, USA). ImageJ software was used to record the optical densities of Western blots and were presented as target protein/β-actin abundance percentage [40].

### 2.15. In Vitro Antiprotozoal Activity of AuNPs and Bionanocomposite

The oil, AuNPs, and bionanocomposite were investigated for in vitro antiprotozoal effect towards *L. amazonensis*, *L. mexicana* promastigotes and *T. cruzi* epimastigotes. The [^3^H] thymidine uptake assay was performed to evaluate growth inhibition of Leishmania spp. promastigotes and *T. cruzi* epimastigotes. The as-prepared AuNPs and bionanocomposite were dissolved in DMSO. The density of cells was adjusted to 1.5 × 10^6^ μg mL^−1^ and cultured in the presence of AuNPs and bionanocomposite at different concentrations (0.1 to 100 μg mL^−1^) for 72 h. Positive controls applied for Leishmania spp. and *T. cruzi* were amphotericin B (0.27–1.6 mM) and benznidazole (5–20 mM), respectively. The inhibition percentage was determined at 48 h by following the equation:

$$\mathrm{Inhibition}\;(\%)=100-\left[\frac{\mathrm{Number}\;\mathrm{of}\;\mathrm{treated}\;\mathrm{parasites}}{\mathrm{Number}\;\mathrm{of}\;\mathrm{untreated}\;\mathrm{parasites}}\right]\;\times \;100$$

The IC_50_ (50% inhibitory concentration) values were calculated by linear regression.

### 2.16. Morphological Changes in Leishmania spp. and T. cruzi

Transmission electron microscopy (TEM) was used to observe the morphological changes in *L. mexicana* promastigotes and *T. cruzi* epimastigotes after treatment with AuNPs and bionanocomposite. *L. mexicana* promastigotes and *T. cruzi* epimastigotes were cultured in either the presence or absence of 2.5 μg mL^−1^ and 10 μg mL^−1^ of AuNPs and bionanocomposite, respectively, for 48 h. For TEM detection, the treated and untreated parasites were washed two times with PBS (pH 7.4). After washing, parasites were centrifuged at 1500 rpm× *g* for 3 min and pellets obtained were kept overnight in cacodylate buffer (pH 7.4) containing 3% glutaraldehyde. Eventually, samples were washed thrice with PBS and post-fixed overnight in 1% osmium tetroxide (OsO_4_). Finally, different concentrations of acetone (50, 75, 90, and 100%) were used to dehydrate the samples and were embedded using the Spurr low-viscosity kit (TED PELLA INC, Redding, CA, USA) overnight at 60 °C. The saturated uranyl acetate was used to stain the ultra-thin sections for 6 min. After staining, the cells were washed again with PBS (pH 7.4) and stained with 5% lead citrate for 4 min. The sections were monitored under Siemens Elmiskop 1 microscope.

### 2.17. Statistical Analysis

Three independent experiments were carried out for each Real-time PCR, cell viability assay, and ELISA measurement. SPSS software (IBM Corporation, Armonk, NY, USA) was used to perform the statistical analysis. Pairwise comparisons were carried out to determine the statistical significance by Kruskal–Wallis one-way ANOVA non-parametric test [41]. Data are expressed as mean ± standard deviation (SD), and the mean difference was calculated at 95% confidence intervals (*p* < 0.05).

## 3. Results and Discussion

### 3.1. GC-MS Analysis of Sesame Oil

**Figure 2:**
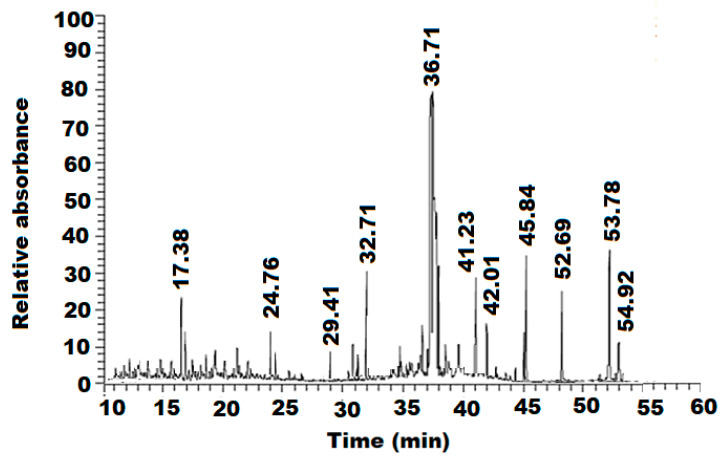
GC-MS chromatogram of ethanol sesame seed extract.

The GC/MS analysis of sesame oil revealed the presence of 11 phytoconstituents, as listed in (Table 2). The identified chemical components were categorized as saturated, monounsaturated, and polyunsaturated fatty acids in 22.04%, 31.54%, and 34.56% proportions, respectively. Cis-9-hexadecenal (35.20%) was found as the major component followed by oleic acid (26.43%) in the oil. Other components present were palmitic acid (8.01%), sesamol (4.52%), and (+)-sesamin (4.91%) (Figure 2).

### 3.2. Characterization of Gold Nanoparticles (AuNPs)

**Figure 3:**
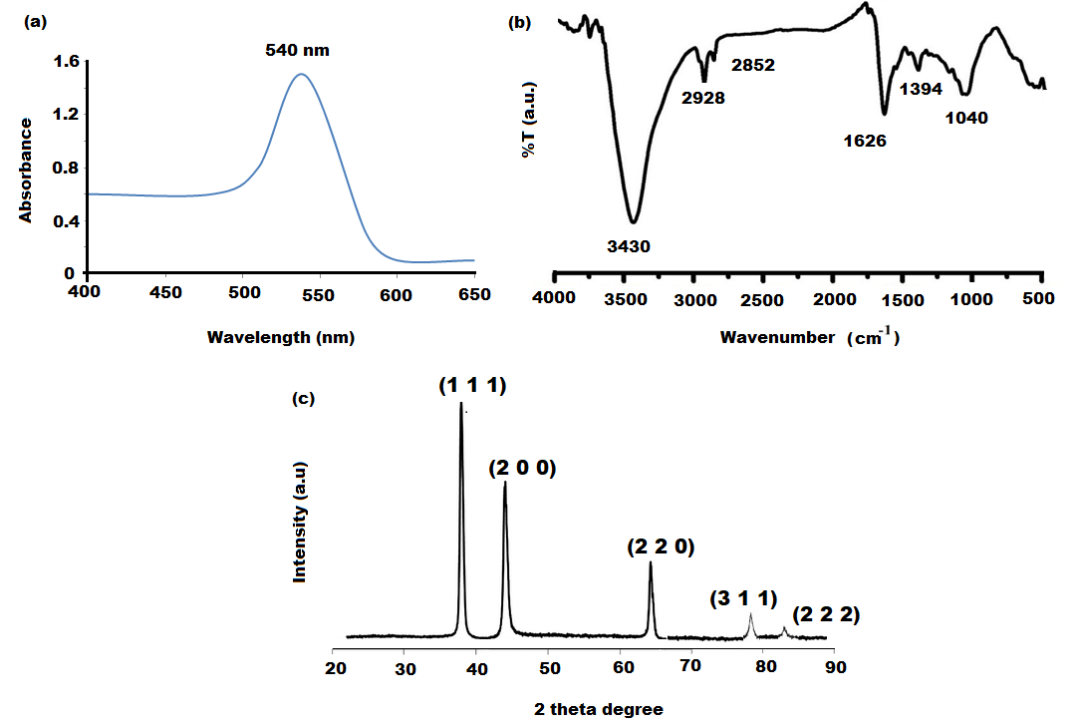
(**a**) UV-Vis, (**b**) FT-IR, and (**c**) XRD spectra of AuNPs.

**Figure 4:**
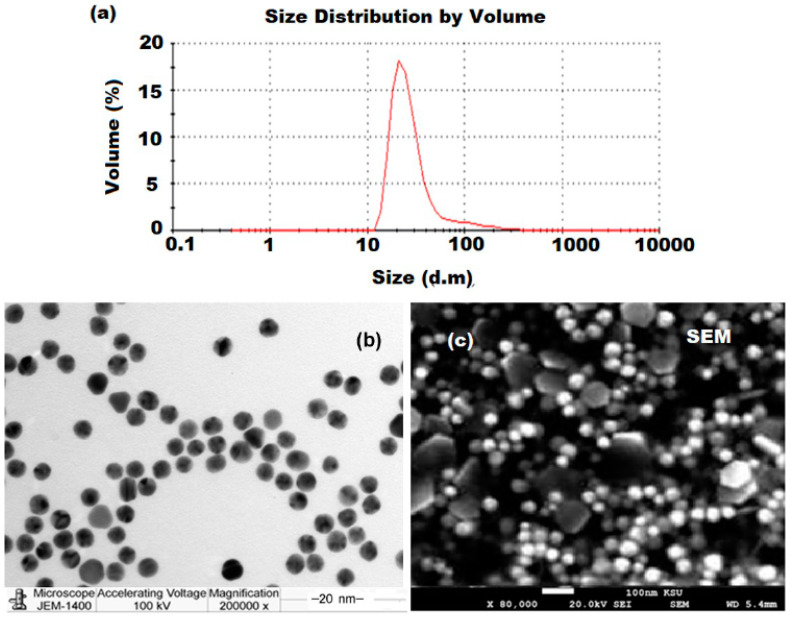
(**a**) DLS, (**b**) TEM, and (**c**) SEM images of AuNPs.

The synthesized AuNPs were characterized by UV-Vis, FTIR, and XRD spectroscopy. The optical characteristic of the as-synthesized AuNPs was studied using UV-Vis spectroscopy. It was observed that AuNPs showed a significant absorption peak at 540-nm wavelength (Figure 3a). A distinct peak revealed the presence of AuNPs with particles’ size of 50 nm, in agreement with the literature [42]. The FT-IR analysis of the prepared AuNPs was performed to ensure their nature and purity. The recorded FT-IR spectrum of AuNPs showed different vibrational peaks, two peaks at 3430 cm^−1^ and 2928 cm^−1^ (O-H symmetric) stretching vibration band. The peak appearing at 1626 cm^−1^ was assigned to the carbonyl (C=O stretching) vibration band due to acetate (COO) of citric acid. Moreover, the vibrational peaks recorded at 1394 cm^−1^ and 1040 cm^−1^ corresponded to the water that adsorbed on the AuNPs’ surface, producing Au–OH and Au* metal nanoparticles (Figure 3b) [43]. The XRD spectrum of AuNPs was recorded to investigate the crystalline characteristics of the synthesized nanoparticles. Four significant peaks, observed at 38.35°, 43.98°, 64.82°, 77.87°, and 82.36°, related to Miller indices Au (1 1 1), Au (2 0 0), Au (2 2 0), Au (3 1 1), and Au (2 2 2), respectively (Figure 3c), were consistent with the literature [44,45]. The presence of these four sharp peaks confirmed the decrease of particle size and the formation of AuNPs. After optimizing the preparation conditions of AuNPs, the crystalline and phase structure were investigated by XRD analysis. The recorded XRD pattern revealed that the formed AuNPs were spherical in shape. The obtained crystal structure was in agreement with the reported face-centered cubic crystalline geometry of AuNPs’ JCPDS file no. 4-0783 [46]. However, the microscopic confirmation was performed using TEM and SEM. The TEM image of the as-synthesized AuNPs indicated that the prepared nanoparticles were uniformly distributed and spherical in shape, with sizes around 20 nm. DLS determined the hydrodynamic diameter or particle size distribution in the solution. The DLS measurement usually represents a bigger particle size than those obtained from TEM and SEM, since the electron microscope gives the determination of particles’ sizes in the dry state. Figure 4a shows the DLS measurement of AuNPs, and the average size of the AuNPs was 50 nm. The DLS measurement displayed a single peak that suggested the homogenous and monodispersed AuNPs in solution with traces of agglomeration. However, agglomeration may happen under storing conditions. The SEM was used to characterize the surface morphology of the formed AuNPs (Figure 4b,c). The sample dissolved in solvent, sonicated for uniform distribution, and directly mounted on the adhesive stub for the SEM investigation.

### 3.3. Characterization of Bionanocomposite

**Figure 5:**
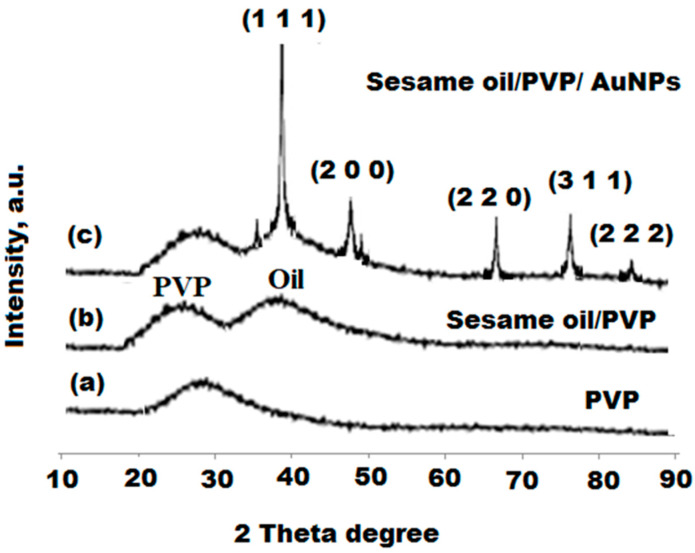
XRD patterns of (**a**) plain PVP, (**b**) sesame oil/PVP, and (**c**) sesame oil oil/PVP/Au bionanocomposite in the range of 20–90 wavenumber.

The polymeric sesame oil/PVP/Au bionanocomposite film was biosynthesized by mixing the extracted sesame oil with a PVP polymer solution and AuNPs at room temperature under continuous stirring. The formation of the sesame oil/PVP/Au bionanocomposite film was confirmed by the color change from a colorless solution to dark purple. Scheme 1 represents the overview procedure for the synthesis of the polymeric sesame oil/PVP/Au bionanocomposite film. The pre-synthesized bionanocomposite was characterized by various techniques including XRD and SEM. The dispersion of sesame oil and AuNPs in the PVP polymeric matrix was confirmed by XRD analysis using Cu kα radiation (λ = 1540 Å) and by applying 30 kV and 60 mA voltage over Bragg angles in the 30° to 90° range. A broad peak appearance in the XRD spectrum of plain PVP indicated its amorphous nature [47] (Figure 5a), whereas the sesame oil showed a high-intensity broad peak near ~30 degrees (Figure 5b). However, the sesame oil/PVP/Au bionanocomposite exhibited distinct peaks of Au (1 1 1, 2 0 0, 2 2 0, 2 2 2, and 3 1 1) at 38.35°, 43.98°, 64.82°, and 77.87°, respectively, in the XRD spectrum (Figure 5c), confirming the excellent distribution of Au nanoparticles above the surface of the bionanocomposite.

**Figure 6:**
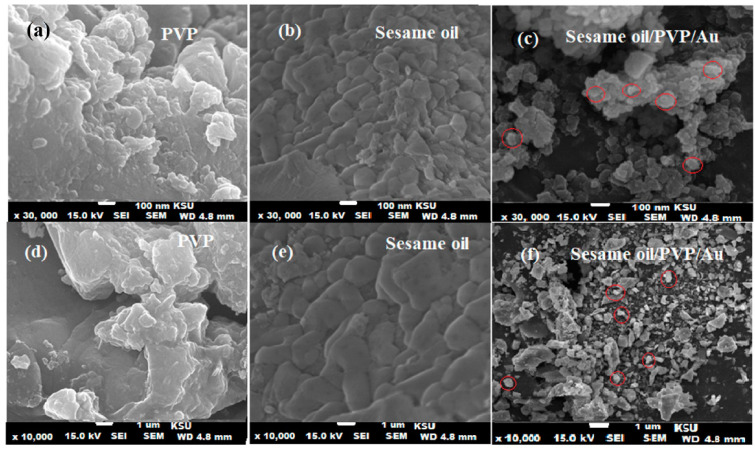
SEM images of (100 nm and 1 μm): (**a**,**d**) plain PVP, (**b**,**e**) Sesame oil, and (**c**,**f**) Sesame oil/PVP/Au bionanocomposite at different, ×10,000 and ×30,000, magnifications.

SEM images were observed at different magnifications to differentiate the plain and formed bionanocomposite. Figure 6a–f demonstrates the surface morphology of PVP, sesame oil, and sesame oil/PVP/Au bionanocomposite. A smooth face with small diameter at 10,000× and 30,000× magnifications represents the PVP (Figure 6a,d). However, the oil droplets were homogeneously distributed with tiny pores on coagulant masses and irregular edges representing the plain sesame oil at the same magnification (Figure 6b,e), whereas the SEM images of the synthesized bionanocomposite expressed well-distributed AuNPs in the PVP polymeric matrix with 50 ± 10-nm particle size under the same magnifications (Figure 6c,f).

**Figure 7:**
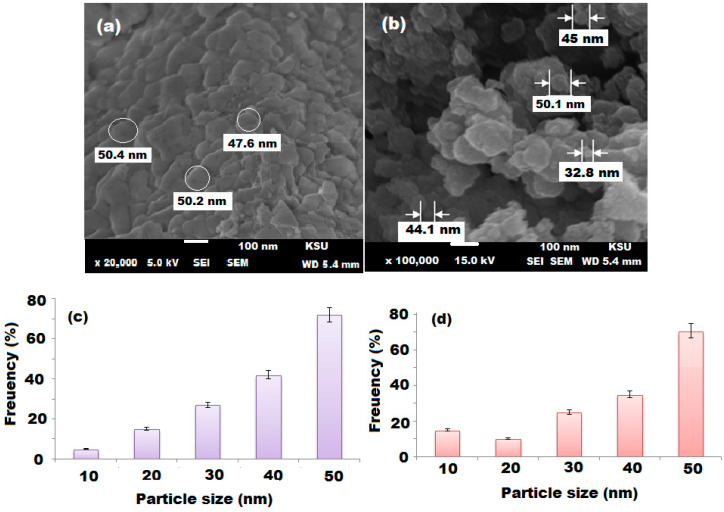
SEM images of (**a**) PVP containing 15 w% of Sesame oil and (**b**) Sesame oil/PVP/Au bionanocomposite. The particle size distribution and diameter were determined for (**a**,**b**) as shown in (**c**,**d**), respectively.

Furthermore, the particle size distribution of PVP polymeric solution containing 10 w % of sesame oil was also examined. The diameter of particle size distribution was found in the range of 40 nm (±10 nm, Figure 7a,c). A homogenous distribution of AuNPs with 47-nm diameter in the polymeric sesame oil/PVP in the sesame oil/PVP/AuNPs bionanocomposite was observed (Figure 7b,d). The uniform surface of the synthesized sesame oil/PVP/AuNPs’ bionanocomposite can be hypothesized by the interaction of AuNPs with sesame oil in the polymeric matrix, thus suggesting the successful utilization of sesame oil with AuNPs in the polymeric PVP medium for the synthesis of the bionanocomposite.

### 3.4. Thermal Stability

**Figure 8:**
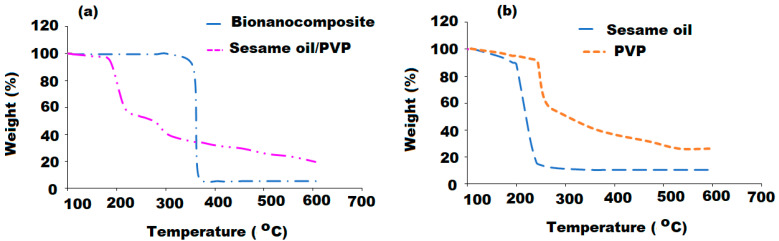
Thermogravimetric analysis of (**a**) Sesame oil/PVP/AuNPs’ bionanocomposite and sesame oil/PVP blend films and (**b**) Sesame oil and PVP.

The thermal stability of sesame oil/PVP, sesame oil/PVP/Au bionanocomposite, and PVP film was examined by TGA, and their TGA and TGAD graphs with those of sesame oil and PVP films for the comparison are shown in Figure 8a,b. The thermal decomposition of the films was monitored by a weight decreasing pattern in the TGA graphs, and the TGAD graphs clearly display the maximum decomposition temperature (*T*_max_) at every step of thermal decomposition. The sesame oil/PVP/AuNPs’ bionanocomposite film exhibited a multistep thermal decomposition due to the thermal decomposition behavior of components of the biogenically synthesized film, i.e., PVP and sesame oil. As demonstrated in the TGA graphs, the process of thermal decomposition of PVP and sesame oil took place in three steps. The initial decomposition of the bionanocomposite film was due to the evaporation of moisture content (10–15%) and total weight loss at around 100 °C. The second thermal decomposition started at 210 °C and 250 °C and provided the maximum degradation rate at 265 °C and 270 °C for the sesame oil and polymeric PVP films, respectively, which was attributed to the volatilization of the components of sesame oil. The main step of decomposition started at 285 °C and 300 °C with the maximum degradation rate at 325 °C and 360 °C mainly attributed to the biopolymeric chain degradation. Presumably, the AuNPs had a dual effect on the bionanocomposite, i.e., catalytic effect towards the polymeric medium and sesame oil that decreased thermal stability and the second as a barrier effect to enhance the thermal stability. The results obtained suggested that the thermal stability of the polymeric bionanocomposite film was up to 360 °C and higher as compared to the individual components due to a strong intermolecular interaction and the inherent features of the samples [48]. Additionally, the interaction between organic and inorganic surfaces of nanomaterials was confirmed by thermogravimetric analysis.

### 3.5. Immunomodulatory Activity

#### 3.5.1. Effects of Nanomaterials on Cell Viability of BV-2 Cells

**Figure 9:**
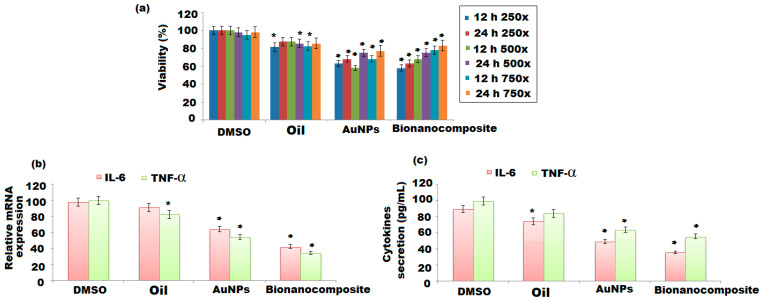
Estimation of cell viability of the treated BV-2 cells. (**a**) CCK-8 cell viability assay for the measurement of BV-2 cells after 12- and 24-h-long treatments with DMSO, sesame oil, AuNPs, and bionanocomposite. Measurement of IL-6 and TNFα levels in mRNA and proteins after sesame oil, AuNPs’, and bionanocomposite treatments of BV-2 cells (**b**) Relative mRNA expression of IL-6 and TNFα of the sesame oil-, AuNPs-, and bionanocomposite-treated cells and (**c**) ELISA measurements of IL-6 and TNFα from supernatant of sesame oil-, AuNPs-, and bionanocomposite-treated BV-2 cells. The mean values and standard deviation (SD) are represented by bars and error bars, respectively, for three independent determinations (*n* = 3); (*) indicate (*p* < 0.05) compared to the control.

The treatment of BV-2 cells with sesame oil, AuNPs, and bionanocomposite might cause adverse effects to the cells; therefore, different concentrations of the test samples (250-fold, 500-fold, and 750-fold) were used to evaluate their effects on cell viability at 12 h and 24 h for long treatments. The carrier used in the stock solution was DMSO, and its effect on the cells was also examined. The results showed no significant effects on the cell viability (Figure 9a). Both the synthesized AuNPs and the bionanocomposite showed significant effects on the BV-2 cells; however, the remarkable alterations in cell viability were exhibited by the biononanocomposite at 12 h in each dilution. Both AuNPs and bionanocomposite enhanced the viability of BV-2 cells at 12 h and 24 h; in the later time point, the increase was significant (Figure 9a). However, sesame oil showed a moderate effect on the cell viability of BV-2 cells. The strong activity of the bionanocomposite could be due to the combined effects of components of sesame oil, AuNPs, and PVP polymeric matrix. As per the obtained results, neither DMSO nor the test samples were toxic for the cells at 24 h. Thus, a 250-fold dilution of samples and standard was selected for further investigations.

#### 3.5.2. Effect of Nanomaterials on mRNA Expression, IL-6, and TNFα Secretion

The increase in the production of proinflammatory cytokines (IL-6 and TNFα) suggests the activation of microglial cells. The BV-2 cells were treated with sesame oil, AuNPs, and bionanocomposite for 24 h to investigate their effect on the levels of mRNA and protein secretions of IL-6 and TNFα cytokines. In comparison to DMSO- and sesame oil-treated cells, both the nanoparticles and bionanocomposite showed a significant decrease in the mRNA expression of IL-6 and TNFα (Figure 9b). The IL-6 secretion was decreased significantly by both AuNPs and bionanocomposite; however, the decrease was more prominent in the bionanocomposite-treated cells (Figure 9c). Furthermore, the results of ELISA measurements revealed that AuNPs and bionanocomposite decreased the secretion of TNFα, indicating that they are the best candidates for the treatment of anti-inflammatory disorder against neuroinflammation (Figure 9c). However, few effects were observed in the expression of IL-6 and TNFα cytokines’ oil-treated samples.

#### 3.5.3. Alteration of mRNA Expression and Secretion of IL-6 and TNFα by Nanomaterials after LPS Pretreatment

**Figure 10:**
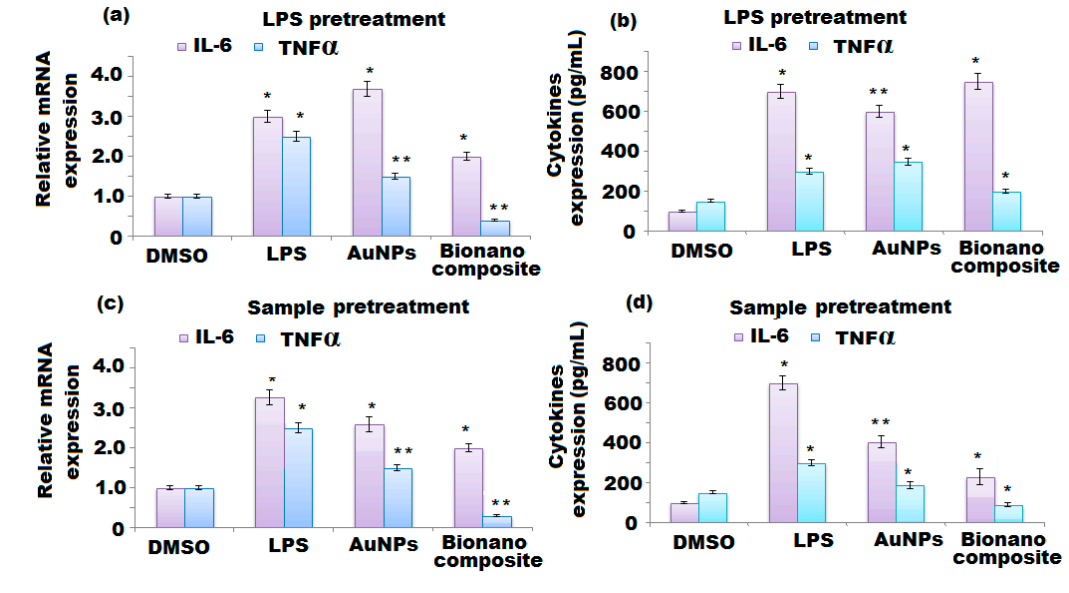
(**a**,**b**) Effect of AuNPs and bionanocomposite on mRNA and protein levels of IL-6 and TNFα after LPS pretreatment. (**c**,**d**) Effect of AuNPs and bionanocomposite after post treatment of LPS. The mean values and standard deviation (SD) are represented as bars and error bars, respectively, in triplicates (*n* = 3); (*) and (**) indicate (*p <* 0.05) compared to the control.

After the inflammation, the BV-2 cells elevated the generation of the proinflammatory cytokines. Experiments were performed to indicate whether AuNPs and bionanocomposite have the ability to improve the secretion of IL-6 and TNFα of BV-2 cells after the 24-h-long LPS pretreatment. AuNPs and bionanocomposite reduced the mRNA level of IL-6, whereas bionanocomposite had the capability to downregulate the IL-6 mRNA expression. However, both AuNPs and bionanocomposite showed a significant decrease in mRNA levels of TNFα expression (Figure 10a). As compared to LPS treatment of BV-2 cells, AuNPs and bionanocomposite were not able to decrease the IL-6 generation, while as, in the case of TNF α cytokine, AuNPs and bionanocomposite potentially decreased the secretion of TNFα. Both AuNPs and bionanocomposite showed potential decrease in the production of IL-6 and TNF α of BV-2 cells; however, the bionanocomposite was found to be more effective (Figure 10b).

#### 3.5.4. Nanomaterials’ Pretreatment Alteration of mRNA Expression and IL-6 and TNFα Secretion of BV-2 Cells Exposed to LPS

Another experiment was conducted to assess whether AuNPs and bionanocomposite pre-treated BV-2 cells have the ability to decrease the inflammatory effect of LPS. The AuNPs and bionanocomposite showed the capability to reduce the levels of IL-6 and TNFα mRNA; however, the bionanocomposite exhibited a strong ability to downregulate IL-6 expression of mRNA. Furthermore, AuNPs and bionanocomposite were found effective only towards the enhanced TNFα mRNA expression (Figure 10c). At the protein level, both AuNPs and bionanocomposite expressed a significant decrease in IL-6 secretion, indicating the delay between mRNA expression and protein synthesis (Figure 10d). The results of TNFα ELISA showed that AuNPs and the bionanocomposite reduced the secretion of TNFα.

#### 3.5.5. Co-Treatment of LPS and Nanomaterials’ Effect on the mRNA Expression and Secretion of IL-6 and TNFα of BV-2 Cells

**Figure 11:**
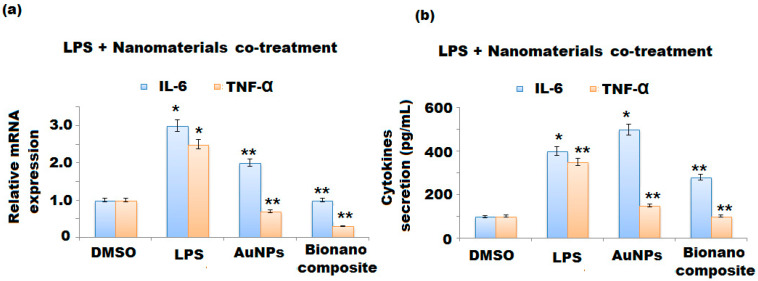
Effect of Co-treatment of LPS and nanomaterials (AuNPs and bionanocomposite) on (**a**) mRNA and (**b**) levels of IL-6 and TNFα. The mean values and standard deviation (SD) are represented as bars and error bars, respectively, in triplicates (*n* = 3); (*) and (**) indicate (*p <* 0.05) compared to the control.

The parallel treatment of BV-2 cells with nanomaterials and LPS was performed to confirm whether the nanomaterials have ability to improve the effect of LPS proinflammatory cytokine production of microglia. The results revealed that AuNPs and the bionanocomposite showed significant potential to decrease the levels of the IL-6 and TNFα cytokines’ mRNA (Figure 11a). Both the nanomaterials were capable of decreasing the secretion levels of IL-6 and TNFα; interestingly, the bionanocomposite expressed a prominent decrease in the secretion levels of cytokines compared to AuNPs in combination with LPS (Figure 11b).

#### 3.5.6. Nanomaterials’ Effect on the NF-κB and Phospho-C/EBPβ Pathways

**Figure 12:**
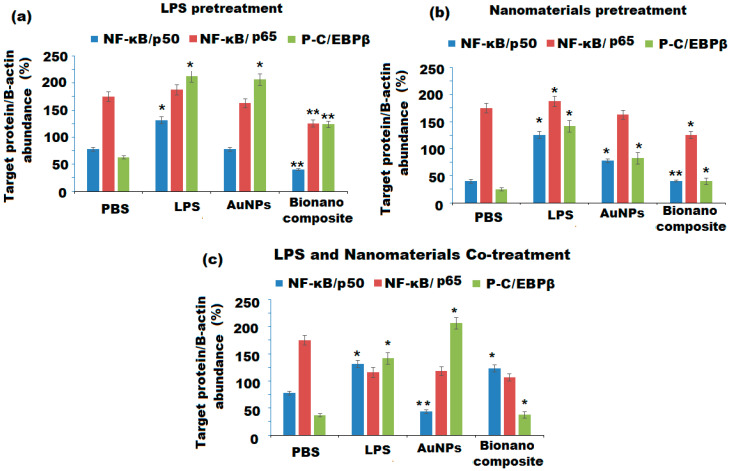
Western blot estimation of NFκB and phospho-C/EBPβ signaling pathways regulating proinflammatory cytokine production of BV-2 cells. Protein levels of p50, p65, and P-C/EBPβ after (**a**) LPS pretreatment, (**b**) nanomaterials’ pretreatment, and (**c**) LPS and nanomaterials’ co-treatment. The mean values and standard deviation (SD) are represented as bars and error bars, respectively, in triplicates (*n* = 3); (*) and (**) indicate (*p <* 0.05) compared to the control.

The NF-κB pathway of microglia was activated by LPS via toll-like receptor 4 (TLR4). The expression of proinflammatory genes (e.g., IL-6 and TNFα) was activated by NF-κB transcription factor after the nuclear translocation by binding to their promoter regions [49,50]. To evaluate the effect of nanomaterials on the protein level of NF-κB, particular antibodies were examined towards p50 and p65 proteins. The LPS pretreatment followed by nanomaterials’ treatment showed the decrease of the p50 level by AuNPs and bionanocomposite, whereas they did not show any significant change in the level of p65 protein (Figure 12a). The pretreatment with nanomaterials partially changed the expression protein levels of NF-κB regulated by LPS. AuNPs reduced the level of p50 and p65 in tested protein. However, the bionanocomposite more decreased the level p50 and p65 compared to AuNPs (Figure 12b). LPS co-treatment with AuNPs and the bionanocomposite exhibited completely different results as compared to the previous, above-mentioned experiment. The bionanocomposite was found to be most effective towards NF-κB proteins; it reduced the levels of all tested proteins. However, AuNPs showed a moderate effect against NF-κB proteins and all the examined proteins (Figure 12c).

As per Western blot results, it appeared that the bionanocomposite had the strongest effect on the LPS-stimulated inflammatory response as compared to AuNPs. Moreover, AuNPs and the bionanocomposite also effectively reduced the effect of LPS treatment on NF-κB pathway. Thus, both these nanomaterials have ability to prevent the activation of NF-κB at LPS treatment. The changes of the NF-κB signaling pathway could not completely reveal the changes that occurred in the expression of TNFα. Therefore, the P-C/EBPβ transcription factor (TNFα regulator) level was also investigated [51]. The results of LPS pretreatments on the P-C/EBPβ transcription factor showed that both AuNPs and the bionanocomposite could attenuate its level (Figure 12a–c).

Recently, inflammation played a key role in the development and seriousness of neurodegenerative disorders such as Parkinson, Alzheimer, and multiple sclerosis diseases. Astrocytes and microglia are responsible for mediating the immunoresponse towards inflammatory agents in the central nervous system [52]. The essential oils are well-known lipophilics and contain organic molecules, which have the ability to transfer across the epithelium and move into systematic circulation to cross the blood–brain barrier and could preserve both neuropathological and behavioral impairments [53,54].

In this study, the potential effects of AuNPs and sesame oil/PVP/AuNPs’ bionanocomposite film in an in vitro microglial cell culture system was investigated. Relatively little information is known about the interaction of nanomaterials and the nervous system cells. Lanza et al. investigated the effects of nanomaterials in a primary rat microglia cell culture and revealed that they serve as a protective agent towards inflammation mediated by LPS administration. According to their study, co-treatment of nanoparticles with LPS to the cultured cells initiated the release of NO, and the expression of inducible NO synthase and cyclooxygenase 2 was decreased by the treatment of nanoparticles [55]. A large number of components are involved in the post-ischemic processes of the brain. Among them, proinflammatory cytokines are the leaders and play a key role in the damage of the blood brain–barrier as well as the activation of microglial cells. BV-2 rodent microglia are commonly used models for evaluating these agents, which may induce the neurodegeneration by microglia activation. LPS is used in the microglia cell culture to initiate the inflammation and for investigating the effect of anti-inflammatory agents [56]. The above study revealed the effects of AuNPs and bionanocomposite on cellular changes at mRNA and protein levels in LPS-treated cells and cells without LPS treatment. Generally, the decrease in secretions and syntheses of IL-6 and TNFα proinflammatory cytokines were observed in the treated cells. A couple of experiments were performed for the comparison: the effects of AuNPs, bionanocomposite, and standards on the BV-2 cells’ survival, the secretion, and synthesis of IL-6 and TNFα. These changes were performed together with LPS treatment in three different ways: LPS pretreatment with nanomaterials, nanomaterials’ pretreatment with LPS, and co-treatment of LPS with nanomaterials. The NF-κB signaling pathway and TNFα regulation effect were evaluated in each of the three treatment versions. IL-6 and TNFα mRNA syntheses and secretions were affected by nanomaterials. It was observed that the cytokine release and mRNA synthesis were not always changing in parallel. The synthesis of protein and the modification of post-translational may be required different regulatory signals than transcription or it is probable that the former processes require more time as compared to mRNA synthesis. The two proinflammatory cytokines (IL-6 and TNFα) showed different secretion patterns with AuNPs and the bionanocomposite. The bionanocomposite displayed a stronger effect in contrast to AuNPs. This phenomenon can be frequently noticed, suggesting that AuNPs can have limitations, whereas the bionanocomposite composed of oil, PVP, and AuNPs has more active components that may act efficiently against inflammation.

Considering alteration in the secretion of IL-6 and TNFα depends on the relation of LPS and nanomaterials’ treatment over time; the best decrease of inflammatory cytokines was noticed by the pretreatment with the nanomaterials. In all these experiments, the reduction of TNFα was higher than that of IL-6 at both the protein and mRNA levels. Additionally, standards had occasionally better effects than nanomaterials, but mainly in mRNA expression. In the nanomaterials’ pretreatment experiments, the bionanocomposite showed outstanding potential. Using essential oil in combination with PVP and AuNPs exhibited excellent results. This might be due to the presence of chemical components in the essential oil that showed a synergistic effect with polymeric nanoparticles and had a specific effect on microglia/macrophages in neuronal injury, hypoxia, and degeneration.

The transcription factor components of NF-κB (chromatin-bound, active p50 and p65) were evaluated to determine the reason behind the changes in the production of proinflammatory cytokines of BV-2 microglia. The activation of transcription of TNFα and IL-6 depends on the action of the heterodimer form of NF-κB [57]. Interestingly, nanomaterials caused a high reduction of chromatin-bound proteins, particularly bionanocomposite, both in the case of pretreatment of LPS as well as pretreatment of nanomaterials’ administration. Moreover, large differences were observed in the levels of P-C/EBPβ after the treatment with standard and nanomaterials (co-treatment and pretreatment) in the cell signaling pathway. Better effects were observed in AuNPs and the bionanocomposite; the latter showed a remarkable effect in the pretreatment. The pronounced immunomodulatory response obtained can be described as the synergistic effect of Au+ ions with sesame oil components, which might have turned the bionanocomposite film to be more active compared to the individual AuNPs and sesame oil. The FTIR spectrum of bionanocomposite also indicated a noticeable alteration in the chemistry of prepared pre-synthesized polymeric bionanocomposite.

### 3.6. In Vitro Antiprotozoal Activity of AuNPs and Bionanocomposite

Leishmanicidal and trypanocidal activities of sesame oil, AuNPs, and bionanocomposite were evaluated by previously described method [58]. The activity of sesame oil and nanomaterials against *L. mexicana* and *L. amazonensis* promastigotes is presented in Table 3. All the tested samples exhibited 50% inhibitory concentration (IC_50_) values less than 20 μgmL^−1^. The best leishmanicidal activity was expressed by bionanocomposite with IC_50_ values of 1.12 ± 1.10 and 1.42 ± 0.69 μgmL^−1^ followed by AuNPs with IC_50_ values of 1.71 ± 1.42 and 1.68 ± 0.75 μgmL^−1^ towards *L. mexicana* and *L. amazonensis*, respectively, whereas sesame oil showed moderate activity against both the parasites. Plant extract oil-incorporated films enhanced antifungal and antimicrobial activity [59,60]. However, the bionanocomposite and AuNPs displayed strong activity against *T. cruzi* epimastigotes with IC_50_ values of 0.05 ± 0.32 and 0.57 ± 0.17 μgmL^−1^, respectively, while sesame oil was found less active (IC_50_ = 2.87 ± 1.28 μgmL^−1^). The incorporation of AuNPs with active phytoconstituents of sesame oil in PVP polymeric medium enhanced the antimicrobial function of the bionanocomposite film. The alleviated antimicrobial effect of the bionanocomposite can be attributed to the combined effect of sesame oil constituents and AuNPs. These active films find more important applications in the food agriculture section to achieve high-quality products and safety [61,62].

### 3.7. Morphological Changes in L. mexicana, L. amazonensis, and T. cruzi

TEM was applied to investigate the morphological alterations and target organelles of *L. mexicana, L. amazonensis, T. cruzi* after the treatment with sesame oil, AuNPs, and bionanocomposite.

**Figure 13:**
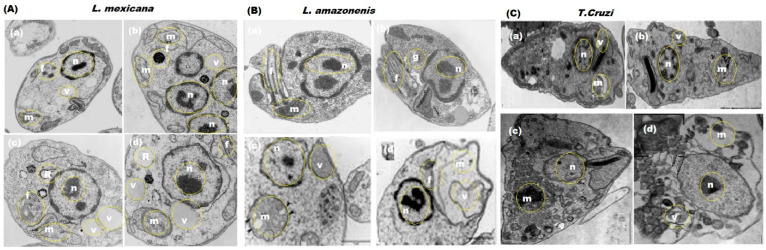
Morphological abnormalities in cultured (**A**) *L. mexicana*, (**B**) *L. amazonensis*, and (**C**) *T. cruzi* after the treatment with (**a**) control, (**b**) sesame oil, (**c**) AuNPs, and (**d**) bionanocomposite at TEM magnification of 4500×. m, v, f, g and R represent mitochondria, vacuole, filaments, golgi bodies, and ribosomes.

After 48-h incubation, intense cytoplasmic vacuolization and destruction of 60% and 75% of the cells were observed in treated *L. mexicana, L. amazonensis, T. cruzi* parasites with AuNPs and bionanocomposite, respectively (Figure 13A–C). Instead, sesame oil induced a mild vacuolization and changes in the shape of all the treated parasites at the ultrastructural level. The enhanced effect of the bionanocomposite compared to AuNPs on tested parasites may suggest that the bionanocomposite possesses a combined effect of oil components and decorated metal nanoparticles on its surface. The decorated AuNPs can easily penetrate the target organelles through the membranes of *L. mexicana*, *L. amazonensis,* and *T. cruzi,* causing the destruction of membranes and releasing the cell cytoplasm as well as cell contents, consequently resulting in parasites’ destruction. Further investigations are required to confirm this hypothesis and to determine such targets.

## 4. Conclusions

The biogenic synthesis of AuNPs and polymeric sesame oil/PVP/AuNP’s’ bionanocomposite using the natural *S. radiatum* seeds oil was described. The prepared AuNPs as well as the polymeric bionanocomposite were characterized by various spectroscopic techniques (UV-Vis, FTIR, XRD) and microscopic techniques (SEM with the EDX and TEM) to confirm their synthesis. The average size of AuNPs was found to be around 20 nm. The SEM micrographs of the bionanocomposite showed well-distributed AuNPs in the PVP polymeric matrix, with a 50 ± 10-nm particle size. The functional groups found in sesame oil as well as AuNPs were confirmed by FT-IR spectrum. Moreover, this study revealed the biogenically synthesized bionanocomposite expressed excellent immunomodulatory and antiprotozoal potentials compared to the individual AuNPs and sesame oil. The anti-inflammatory effect of AuNPs and the polymeric bionanocomposite on LPS-induced BV-2 microglia revealed that both the nanomaterials exhibited anti-inflammatory effect on LPS-induced microglia via modulating the activation of NF-κB and C/EBPβ signaling pathways and lowering the secretion of proinflammatory cytokines (IL-6 and TNFα). The outcomes showed that the bionanocomposite exerted a higher inhibitory effect on the tested signaling proteins. Based on these results, the bionanocomposite can serve as complementary neurotherapeutics towards neuroinflammation. The results of the current study favor the utilization of sesame oil as a natural source for the treatment of inflammation. Additionally, the polymeric bionanocomposite showed potent antiprotozoal activity against *L. mexicana, L. amazonensis, and T. cruzi* parasites.

## Figures and Tables

**Table 1:** List of gene primer by PCR real time.

Primer	$\mathrm{Sequence}\;5^{\prime }\to 3^{\prime }$
IL-6 forward	CTCTGCAAGAGACTTCCATCCA
IL-6 reverse	GACAGGTCTGTTGGGAGTGG
TNFα forward	GATCGGTCCCCAAAGGGATG
TNFα reverse	CCACTTGGTGGTTTGTGAGTG
β-actin forward	CTGTCGAGTCGCGTCCA
β-actin reverse	TCATCCATGGCGAACTGGTG

**Table 2:** The determined active components in the ethanol extract of sesame seeds using GC-MS.

No.	Name of Compound	Rt	Peak * Area %	Molecular Formula	Molecular Mass	Nature
1	Carvacrol	16.70	1.05	C_10_H_14_O	150.22	Phenol
2	Sesamol	17.44	4.52	C_7_H_6_O_3_	138.12	Phenol
3	Eicosanoic acid methyl ester	24.67	0.21	C_21_H_42_O_2_	326.56	Fatty acid
4	9-hydroxy pentadecanoic acid-methyl ester	24.76	0.14	C_19_H_38_O_3_	314.50	Fatty acid
5	Docosanoic acid methyl ester	29.41	0.12	C_23_H_46_O_2_	354.61	Fatty acid
6	Palmitic acid	32.71	8.01	C_16_H_32_ O_2_	256.43	Fatty acid
7	Cis-9-hexadecenal	36.71	35.2	C_16_H_30_O	238.41	Fat
8	Oleic acid	41.25	26.43	C_18_H_34_O_2_	282.47	Fatty acid
9	Palmitic acid-β- monoglyceride	42.01	0.5	C_19_H_31_ClO	330.5	Fat
10	Oleoyl chloride	45.84	1.2	C_18_H_33_ClO	300.9	Salt of fatty acid
11	Methyl ricinoleate	51.26		C_19_H_36_O_2_	312.5	Salt of fatty acid
12	(+)-Sesamin	52.69	4.91	C_20_H_18_O_6_	354.35	Lignan
13	6-Nitrocholest-5-en-3-yl acetate	53.78	0.32	C_29_H_47_NO_4_	473.7	Steroid
14	Propylidenecholesterol	54.89	0.21	C_30_H_50_O	480.6	Steroid
15	Ergost-5-en-3β-ol	54.92	1.95	C_28_H_48_O	400.7	Steroid

* The peak area percentage was calculated by applying the following formula: Peak area (%) = (Area of the peak)/(total area) × 100%.

**Table 3:** In vitro antiprotozoal activity of sesame oil, AuNPs, and bionanocomposite against *L. mexicana*, *L. amazonensis* promastigotes *and T. cruzi* epimastigotes.

IC_50_ (µg mL^−1^)			
Sample	*L. mexicana*	*L. amazonensis*	*T. cruzi*
Sesame oil	5.31 ± 1.22	8.84 ± 2.21	2.87 ± 1.28
AuNPs	1.71 ± 1.42	1.68 ± 0.75	0.57 ± 0.32
Bionanocomposite	1.12 ± 1.10	1.42 ± 0.69	0.05 ± 0.17

## Data Availability

The data support the findings of this study are included within the text.
